# Comparison of feature point detectors for multimodal image registration in plant phenotyping

**DOI:** 10.1371/journal.pone.0221203

**Published:** 2019-09-30

**Authors:** Michael Henke, Astrid Junker, Kerstin Neumann, Thomas Altmann, Evgeny Gladilin

**Affiliations:** Leibniz Institute of Plant Genetics and Crop Plant Research (IPK Gatersleben), OT Gatersleben, Corrensstr. 3, D-06466 Stadt Seeland, Germany; University of Helsinki, FINLAND

## Abstract

With the introduction of multi-camera systems in modern plant phenotyping new opportunities for combined multimodal image analysis emerge. Visible light (VIS), fluorescence (FLU) and near-infrared images enable scientists to study different plant traits based on optical appearance, biochemical composition and nutrition status. A straightforward analysis of high-throughput image data is hampered by a number of natural and technical factors including large variability of plant appearance, inhomogeneous illumination, shadows and reflections in the background regions. Consequently, automated segmentation of plant images represents a big challenge and often requires an extensive human-machine interaction. Combined analysis of different image modalities may enable automatisation of plant segmentation in “difficult” image modalities such as VIS images by utilising the results of segmentation of image modalities that exhibit higher contrast between plant and background, i.e. FLU images. For efficient segmentation and detection of diverse plant structures (i.e. leaf tips, flowers), image registration techniques based on feature point (FP) matching are of particular interest. However, finding reliable feature points and point pairs for differently structured plant species in multimodal images can be challenging. To address this task in a general manner, different feature point detectors should be considered. Here, a comparison of seven different feature point detectors for automated registration of VIS and FLU plant images is performed. Our experimental results show that straightforward image registration using FP detectors is prone to errors due to too large structural difference between FLU and VIS modalities. We show that structural image enhancement such as background filtering and edge image transformation significantly improves performance of FP algorithms. To overcome the limitations of single FP detectors, combination of different FP methods is suggested. We demonstrate application of our enhanced FP approach for automated registration of a large amount of FLU/VIS images of developing plant species acquired from high-throughput phenotyping experiments.

## Introduction

With the rise of high-throughput multi-camera systems during the past decades, modern phenotyping facilities provide biologists with ever growing amount of multimodal image data. The visible light spectrum (VIS) and fluorescence (FLU) images are two most frequently used image modalities to assess optical plant appearance and their chlorophyll content. The first step towards quantitative analysis of plant image consists in finding of relevant plant structures (i.e. image segmentation) which is often hampered by a low contrast between plant and non-plant image regions. A straightforward segmentation of plant structures using simple intensity-thresholding techniques is not feasible because of variable scene illumination, shadows, reflections and partially overlapping plant and background colours. Consequently, additional spatial information is required for reliable segmentation of plant structures. For this purpose, combination of different image modalities can be used. In order to perform a combined analysis of FLU and VIS images taken by cameras of different spatial resolution from different positions they have to be geometrically aligned. In previous works, manual registration of one test FLU/VIS image pair was suggested for derivation of a relative geometric transformation which is then applied for all subsequent images of the same experiment [1]. However, due to a number of factors such as daytime variation of room temperature, different plant sizes, as well as different varying distances between camera and plants for different rotation angles geometric transformations required for FLU/VIS image registration undergo changes in course of times. Consequently, every FLU/VIS image pair has to be ideally aligned anew.

Automated image registration aims to calculate relative geometric transformations between each two images in a fully unsupervised manner. In case when images of different modalities are acquired simultaneously, alignment of images acquired with different optical sensors can be performed using calibration tables [2, 3]. However, in Greenhouse phenotyping systems like ours FLU and VIS images are acquired sequentially in different, spatially separated photo-chambers. Consequently, calibration of FLU/VIS cameras by means of multimodal calibration tables is not possible in our setup. Instead, correspondences and geometric transformations between FLU and VIS images have to be found algorithmically using suitable silent image features.

In previous literature, different rigid and non-rigid registration approaches including feature-point (FP), optical flow (OF), frequency-domain (FD) and intensity information (INT) methods have been used for establishment of image correspondences and alignment [4–8]. Image registration was frequently applied for alignment and fusion of medical, microscopic and aerial images. Applications of image registration in context of multimodal plant image analysis are, however, relatively scarce [9, 10].

Differently from multimodal medical images (e.g., CT, MRI), FLU and VIS images of plant shoots exhibit local structural similarity which can be detected by means of feature point detectors. FP detection represents a particularly interesting method for plant image analysis as it can be used not only for registration but also for identification of plant structures such as leaf tips, flowers, etc. In view of a large amount of image data (10^5^−10^6^ images per each experiment), simultaneous detection of relevant plant structures and utilization of this information for multimodal image registration is of immediate advantage for algorithmic performance.

For establishment of pair-wise image correspondences different image features can be used. While some FP methods try to identify image edges or corners others rely on local intensity information, for example, by constructing structure tensors or statistical descriptors. Common feature detection approaches are: point, edge and corner detection (e.g., FAST [11], Shi and Tomasi [12], Harris operator [13], SUSAN [14]), blob detection (e.g., MSER [15], DoG, DoH), structure tensors, and feature description (e.g., SURF [16], HOG, SIFT [17]). Most of these methods are not invariant to photometric distortions in contrast and brightness and, therefore, are sensitive to statistical and structural noise leading to a large amount of non-matching feature points. For a detailed introduction and comparisons of further feature detectors we refer to [18–21].

**Fig 1:**
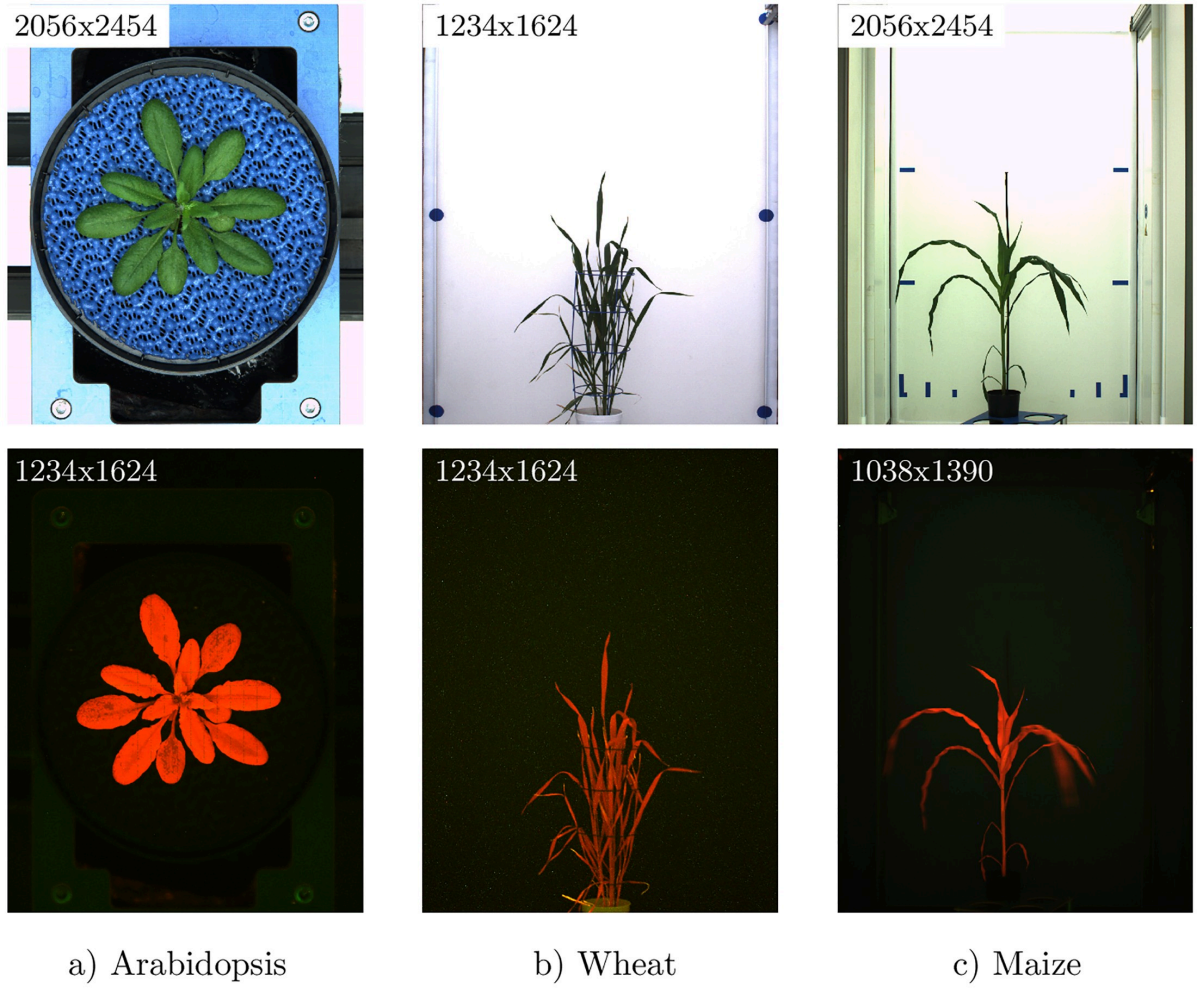
Example images for the three experiments (Arabidopsis, wheat, and maize) of late developmental stages in visible light (top) and corresponding fluorescence images at the bottom side.

Detection of reliable feature points and matching FP pairs is known to be sensitive to differences in background structures and modalities-specific noise. For example, contours of walls, carriers and other light reflecting/absorbing objects in VIS images are typically not visible in FLU images that mainly show chlorophyll containing plant structures, see Fig 1. Due to non-homogeneous illumination, shadows and reflections, background and plant regions of VIS images undergo considerable variations in intensity and colour. Furthermore, plant images can exhibit non-uniform image motion (for example, caused by inertial movements of leaves after relocation of plants from one photo-chamber to another) which additionally complicates a straightforward image registration. To enhance similarity between images of different modalities and to enable robust and accurate detection of relevant plant structures, suitable image pre-processing is required. Here, we investigate effects of background filtering by comparing the results of FP registration of original intensity (i.e. unfiltered)vs. manually segmented, cropped and gradient (i.e. edge) images.

In view of a large variability in optical appearance of plant structures ranging from round Arabidopsis leaves in top view to thin, curved leaves of wheat or maize shoots in side view (see Fig 1), one particular FP detector would unlikely be suitable for detection of all possible plant phenotypes. Consequently, it is reasonable to approach the problem of multimodal plant image registration in a very general manner by combining advantages of multiple FP detectors. Our experimental results show that by combination of appropriately adjusted FP detectors fully automated co-registration of heterogeneous FLU and VIS plant images can be performed.

## Materials and methods

### Feature detection

In this study, the MATLAB (MathWorks, Inc.) computing environment was used to compare the performance of seven different algorithms for automated feature detection and their usability in multimodal image registration of plant images. The MATLAB Image Processing Toolbox (2018a) provides a set of seven feature detection methods, see Table 1.

**Table 1:** Overview of FP detection functions distinguished on used feature types provided by the MATLAB Image Processing Toolbox (2018a).

Detector	Features	Function	Literature
**BRISK**	Corners	*detectBRISKFeatures*	[22]
**FAST**	Corners	*detectFASTFeatures*	[11]
**Harris**	Corners	*detectHarrisFeatures*	[13]
**KAZE**	Blobs	*detectKAZEFeatures*	[23]
**MinEigen**	Corners	*detectMinEigenFeatures*	[12]
**MSER**	Blobs	*detectMSERFeatures*	[15]
**SURF**	Blobs	*detectSURFFeatures*	[16]

### Plant material

The plant image material used within this study consists of example images of three independent experiments carried out at the IPK Gatersleben between 2015 and 2016. The image material was obtained by three different high-throughput facilities (Greenhouse Scanalyzer3D Systems, https://lemnatec.com/products/greenhouse-scanalyzer-system/greenhouse-scanalyzer/) made by LemnaTec company (LemnaTec GmbH, Aachen, Germany), each with its own configuration and specialisations for small, medium size and large plants.

In the highest expansion stage, the LemnaTec Scanalyzer3D consists of three measuring boxes, each equipped with one (or more) different sensor systems. Following a measuring plan, plants are removed automatically from the greenhouse to the measuring facility where they are successively channelled from one measuring box to the next one. Corresponding VIS and FLU images are therefore taken within a time span of a few seconds, which is required to relocate the plants. The experiments were chosen to reflect the large variety in optical conditions and facility set-ups, see Fig 1. From 10^5^−10^6^ images acquired by every phenotyping experiment, a representative subset of totally 1170 FLU/VIS image pairs was selected for manual segmentation and FP algorithm evaluation. This test dataset includes three species (Arabidopsis, wheat and maize) measured at different developmental stages in top/side views and different rotation angles, Table 2.

**Table 2:** The data set used in this study consists of three different experiments including three different species of top and side view images, each taken in visible light and fluorescence, obtained by three independent LemnaTec facilities for large intermediate size and small plants at the IPK Gatersleben.

Spicies (view)	# Plants	# Days	# Angles	# FLU/VIS pairs	VIS size	FLU size
Arabidopsis (top)	4	20	1	80	2056x2454	1234x1624
Wheat (side)	4	47	3	564	1234x1624	1234x1624
Maize (side)	6	22	4	528	2056x2454	1038x1390

### Image pre-processing

From previous studies and from the literature [4], it is known that detection of corresponding feature points is particularly difficult for non-identical multimodal images.

**Fig 2:**
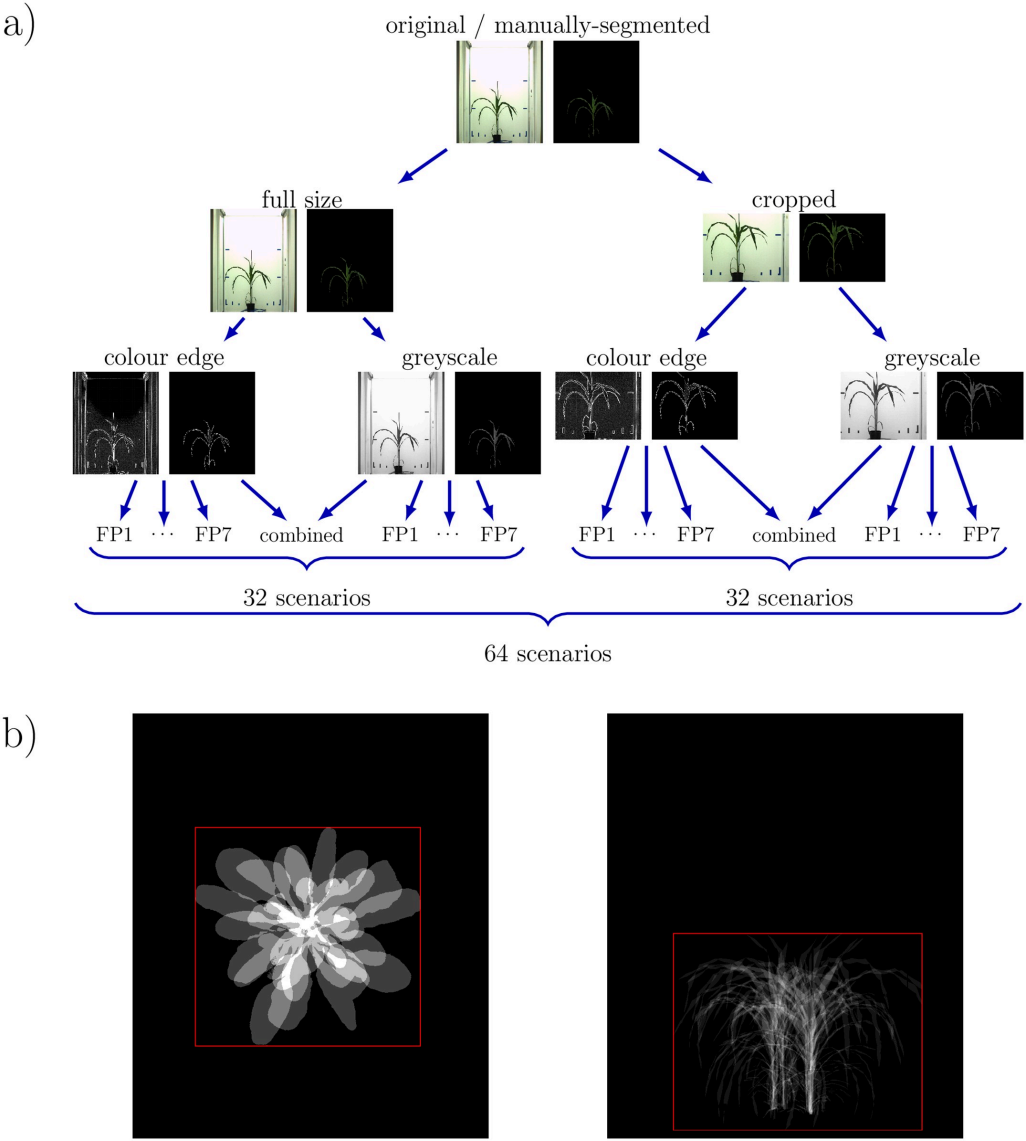
Overview of investigated image pre-processing scenarios. (a) In addition to original intensity images, full size and cropped manually segmented and colour edge images were used. All eight FP detectors (seven single plus one combined FP methods) were applied to each and every original and pre-processed FLU/VIS image pair as well as the combination of different FP detectors, resulting in totally 64 different pre-processing/registration tests. (b) The maximum bounding box used for cropping of plant regions of each age class was obtained by overlapping of all plants of the same age (here for two cases: left for Arabidopsis top view, and right for maize side view).

In order to investigate the effects of structural image improvement three different pre-processing conditions and their combinations have been introduced. These conditions include manual image segmentation, image cropping and colour-edge transformation, see an overview in Fig 2(a).

The manual image segmentation (MS) (“ground truth”) corresponds to an ideal background filtering. It was introduced as a reference to original (i.e. unfiltered) intensity images to study effects of background elimination on the results of image registration. Image cropping, i.e. reduction of the image size to the bounding box of the plant, also largely eliminates non-plant structures which makes FLU/VIS more similar. For cropping of the plant regions the maximum bounding box of all pre-segmented plants of the same age (i.e. experiment day) was calculated, see Fig 2(b).

Third tested pre-processing condition consists in calculation of colour-edges for the given pair of FLU/VIS intensity images [24]. Colour-edges eliminate large range inhomogeneity of image intensity distribution such as the vertical gradient of background illumination in VIS images, which effectively increases structurally similarity between FLU and VIS images.

### Evaluation of FP registration and effects of image pre-processing

All eight FP detectors (seven single plus one combined FP methods) were evaluated with different combinations of pre-processing steps including original vs manually segmented (see Table 1), greyscale vs colour-edge, and full-size vs cropped images, resulting in totally 64 = 2 ⋅ 2 ⋅ (2 ⋅ (7 + 1)) cases per each FLU/VIS image pair (Fig 2(a)).

**Fig 3:**
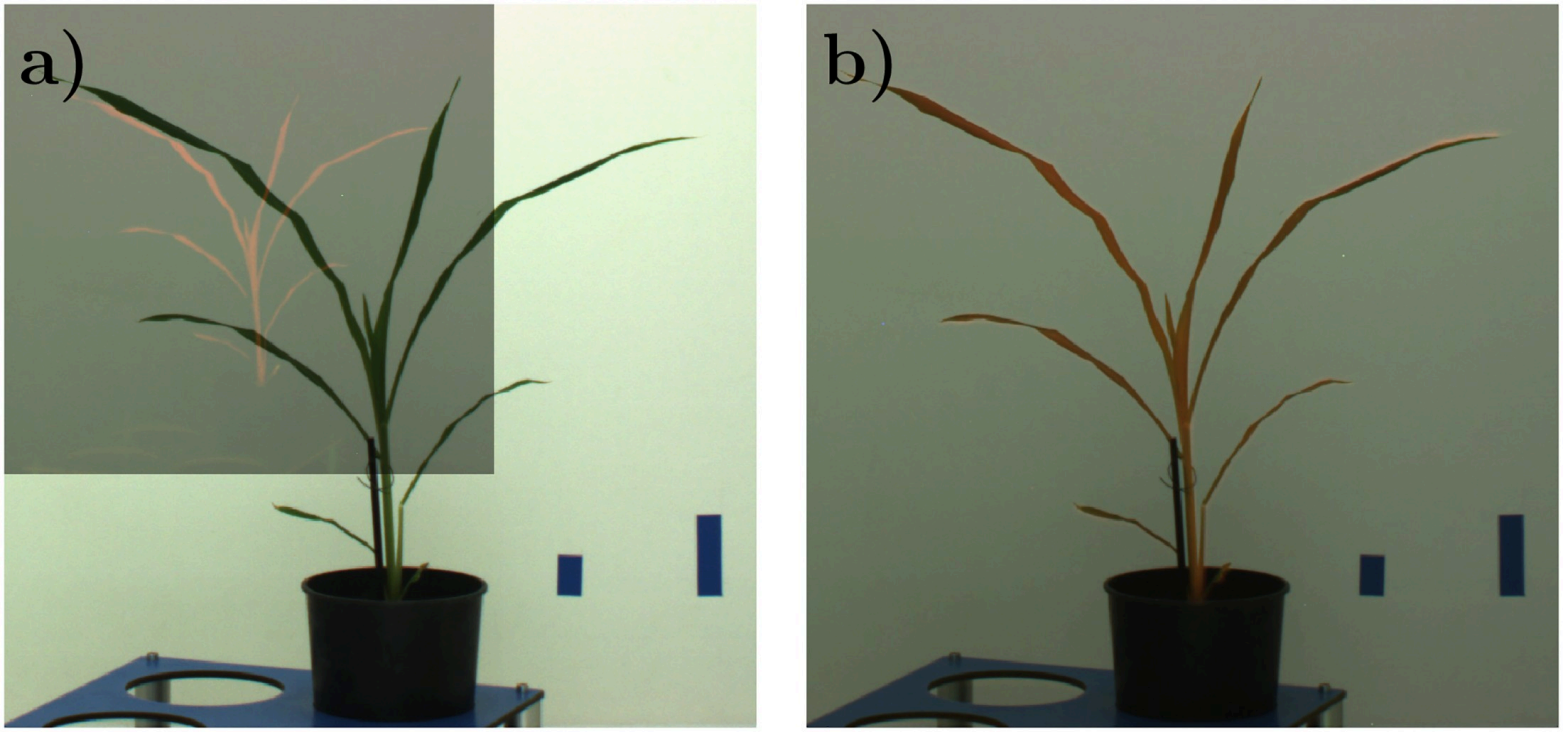
Example of registration FLU/VIS images of a young maize shoot. (a) Overlap of originally- sized FLU/VIS images. (b) The result of successful FLU/VIS image registration using SURF FP detector.

The results of image registration were evaluated in terms of (i) reliability of acquired transformation matrix (i.e. the binary yes/no criterion) and in terms of (ii) accuracy quantified by the amount of overlapping area between registered FLU and manually segmented VIS images. Fig 3(a) shows an example of FLU/VIS images of a young maize shoot image in original resolution. The result of a successful registration, where the FLU was mapped onto VIS image, is shown in Fig 3(b).

**Fig 4:**
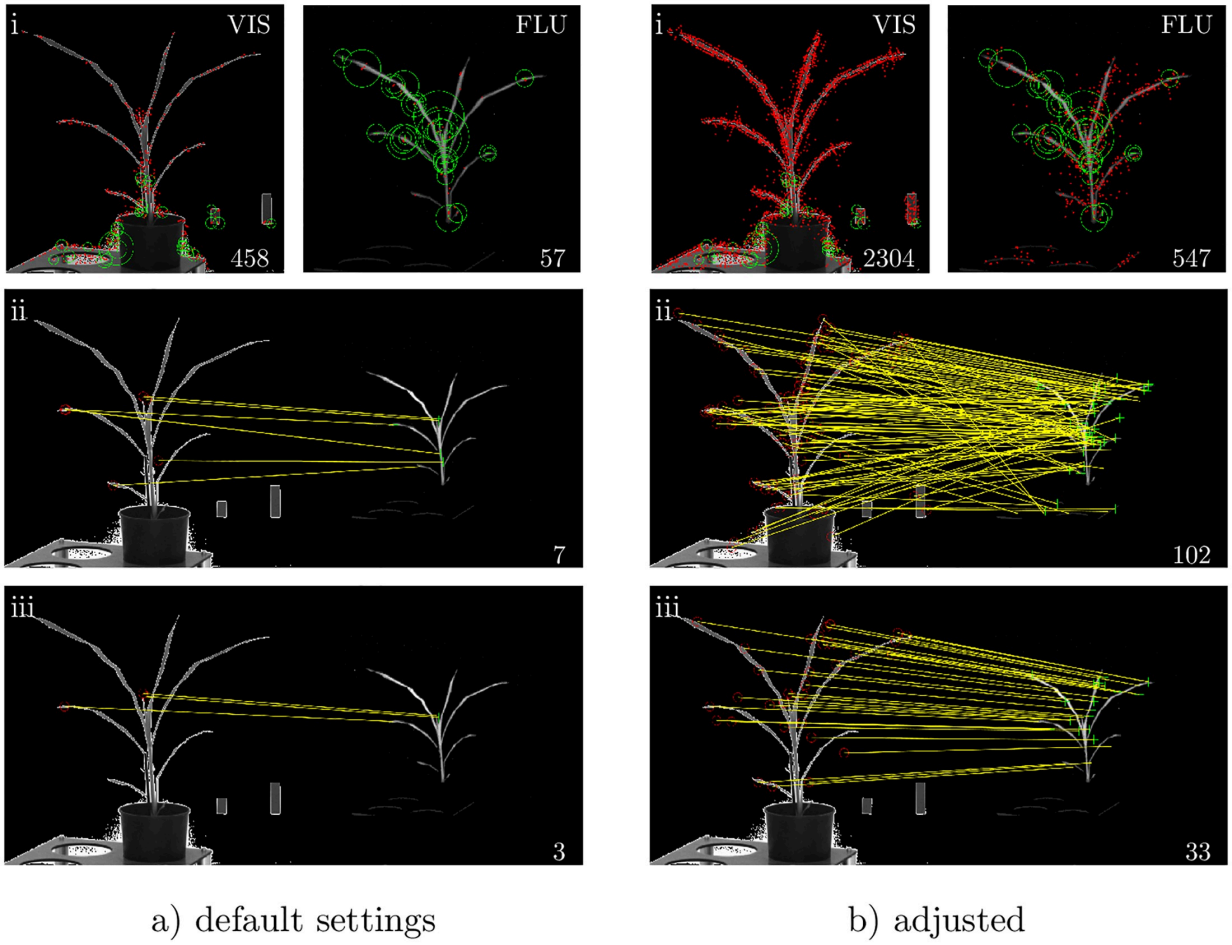
Example of the step-wise process of SURF FP selection and evaluation for a pair of FLU/VIS maize images using the MATLAB default parameter settings (a) and our adjusted set of parameters (b). Figures in top row (i) illustrate the initial set of feature points (red dots) for VIS and FLU including the 30 points with the strongest SURF metric (green dots). The total number of detected FPs/point pairs is given at the lower right corner. Figures in the middle row (ii) show the putative matching of FLU/VIS feature points (highlighted by connecting yellow lines). In the bottom row (iii), the final pairs of FLU/VIS feature points selected for calculation of FLU/VIS image transformation are shown.

Fig 4 illustrates the basic steps of image registration using FP matching for MATLAB default settings (a) and adjusted settings (b). First, an initial set of feature points is pre-selected using one of the FP detectors (in this example, SURF) for each FLU and VIS image separately, Fig 4(i). In the second step, pre-selected feature points in FLU and VIS images are evaluated according to the squared *L*_2_-norm (sum of squared difference) between specific feature vectors of a particular FP detector, Fig 4(ii). Only pairs of feature points with the distance measure below a certain threshold are accepted for establishment of image correspondences. At this stage, the matching pairs of feature points may include inconsistent correspondences that do not allow calculation of a reliable affine transformation. Inconsistent pairs of FP points are then detected and excluded by means of an RANSAC optimisation as described in [25]. Consequently, the actual image transformation is calculated using a significantly reduced set of pair-wise FLU/VIS correspondences Fig 4(iii).

To enable generation of a sufficient number of feature points and their putatively matching pairs in non-identical FLU/VIS plant images, default parameters of MATLAB FP registration routines need to be adjusted. Following the MATLAB’s API, default parameters of FP-detection, -matching and transformation estimation routines were amended to increase (i) the number of detected feature points, (ii) accepted putatively matching FP pairs, and (iii) to improve robustness of calculation of geometric transformations. Fig 4(a) and 4(b) illustrate the difference between the MATLAB default parameter settings and our adjusted set of parameters, respectively.

As one can see adjustment of parameters results in substantial increase of the number of initially detected as well as finally approved FP pairs. Table 3 gives an overview of parameters that were adjusted for better performance of FP routines in comparison to default MATLAB values.

**Table 3:** Default vs adjusted parameters of MATALB FP registration routines.

MATLAB routine	Parameter name	Default value	Adjusted value	Effect of parameter adjustment
*detectBRISKFeatures*	MinContrast	0.2	0.1	reduce the number of detected corners
*detectFASTFeatures*	MinContrast	0.2	0.1	reduce the number of detected corners
*detectHarrisFeatures*	FilterSize	5	3	increases number of FPs
	MinQuality	0.01	0.03	remove erroneous corners
*detectKAZEFeatures*	NumOctaves	3	4	increases number of FPs
	NumScaleLevels	4	6	increases number of FPs
	Threshold	0.01	0.02	exclude less significant local extrema
*detectMSERFeatures*	ThresholdDelta	2	0.8	return more regions
*detectMinEigenFeatures*	FilterSize	5	3	increases number of FPs
	MinQuality	0.01	0.03	remove erroneous corners
*detectSURFFeatures*	NumOctaves	3	4	detect larger blobs
	MetricThreshold	1000	25	increases number of returned blobs
*matchFeatures*	MatchThreshold	10	15	return more matches
	MaxRatio	0.6	0.75	return more matches
	Unique	false	true	only unique FP pairs allowed
*estimateGeometricTransform*	MaxDistance	1.5	10	decreases the accuracy
	Confidence	99	95	downgrade the robustness of the results
	MaxNumTrials	1000	5000	improves the robustness of the results

#### Criteria of registration robustness and accuracy

For assessment of robustness and accuracy of the image alignment, two following criteria were introduced. The first criterion—the success rate (*SR*) of image registration is calculated as the ratio between the number of successfully performed image registrations (*n*_*s*_) divided by the total number of registered image pairs (*n*):

$$\begin{array}{c} SR=\frac{n_{s}}{n}. \end{array}$$ (1)

As a criterion of success of image registration, min/max bounds for admissible scaling, rotation and translation were defined. Transformations that do not fit into these bounds were treated as failure of image registration.

The second criterion is constructed to quantify the overlap ratio (*OR*) between the area of plant regions in VIS images that are covered by the registered FLU image (*a*_*r*_) and the total area of manually segmented (“ground truth”) plant regions in VIS images (*a*):

$$\begin{array}{c} OR=\frac{a_{r}}{a}. \end{array}$$ (2)

While *SR* serves as a criterion indicating that registration routine succeed in producing some reasonable transformation, *OR* describes the accuracy of successful registration.

## Results

**Fig 5:**
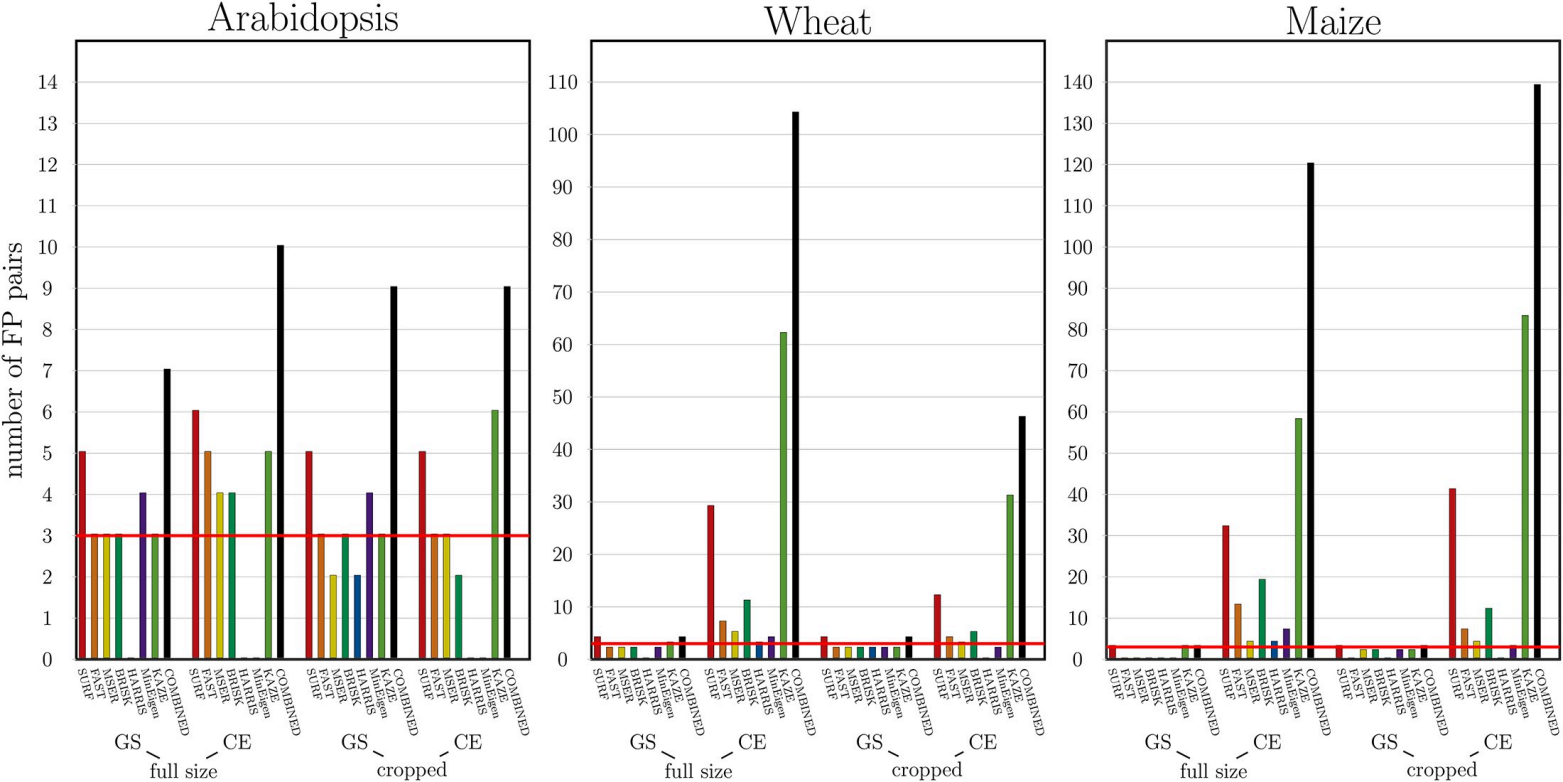
Statistics of average number of pairs of feature points finally used for registration for each FP detector and for the combined detector for successful registrations of original images in full size and cropped (the “S”-column in Table 4). As the data show, most single FP detectors in average do not produce the minimum number of three pairs which is required for an affine registration (red horizontal line). The combined detector (black bar) on the other hand can help to increase the FP number to a sufficient amount.

**Fig 6:**
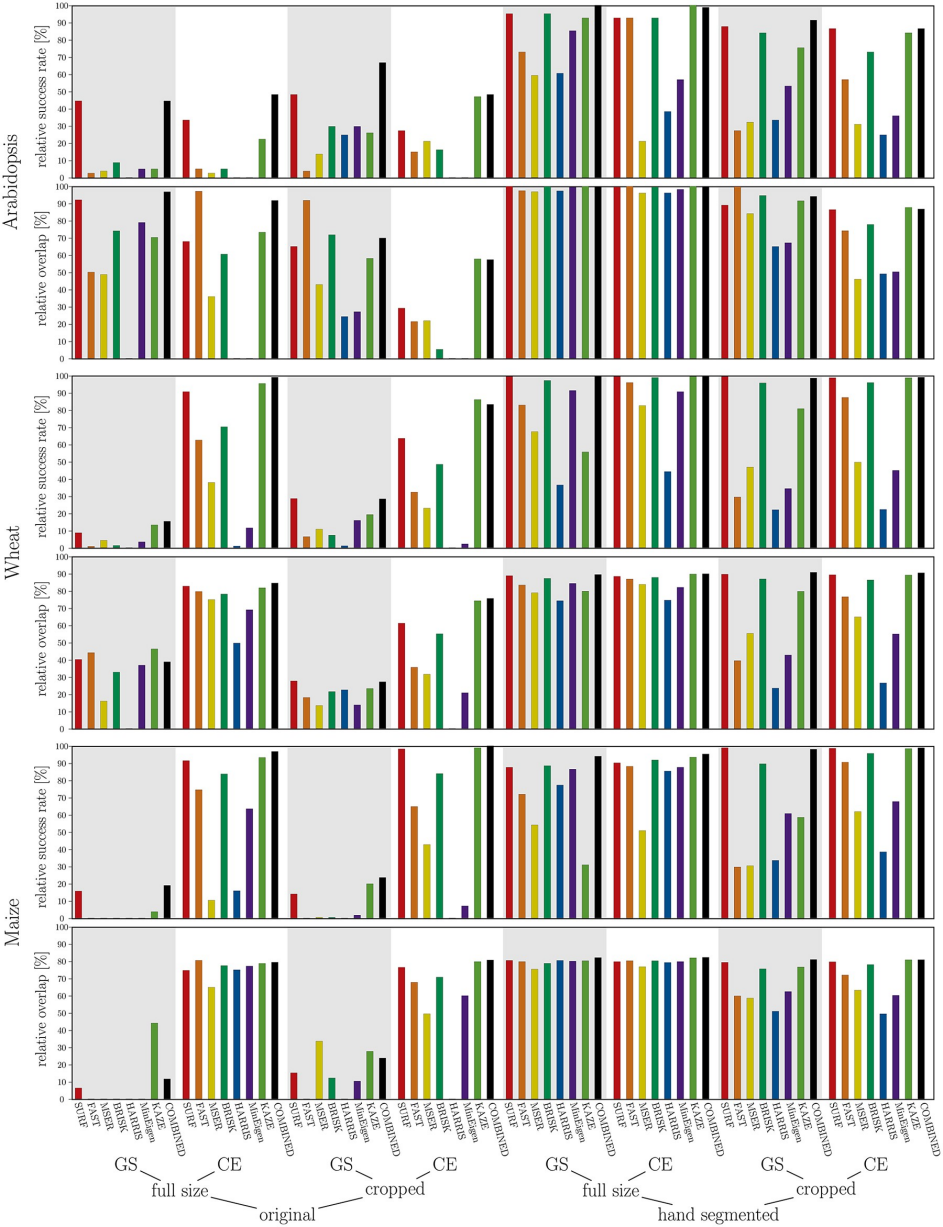
Success rate (*SR*) and overlap ratio (*OR*) of FP registration of original and manually segmented maize shoot images in greyscale and colour-edge modes for all feature point detection methods individually as well as for the combined feature point detector for each image pre-processing class (black-coloured bar).

First, the performance of seven feature point detectors by registration of original (i.e. greyscale-transformed, but otherwise unchanged) FLU and VIS plant images was evaluated. For this purpose, the number of the detected feature points, putatively matching and finally accepted FP pairs as well as the success rate and the overlap ratios of single FP detectors was assessed. The results of these tests showed that all seven FP algorithms exhibit unsatisfactory performance using the MATLAB default set of parameters. While some FP detectors (e.g., SURF, KAZE) were initially capable to find more feature points than the others (see Table 4, Fig 5), the final number of putatively matching FP pairs remained very low. Often, not a single FP pair out of thousands of initially detected feature points was approved to be reliable which results in the total failure of FP registration. Consequently, the average success ratio of FP registration algorithms using the MATLAB default parameters lays below 0.5, see Fig 6. As exemplary shown in Fig 4(a), from originally detected 458 SURF points in VIS and 57 in FLU images of young maize plants, 7 putatively matching FP pairs were found to be reliable. However, from the 7 FP pairs only 3 (the actual minimum of the required number of FP points) were finally accepted for calculation of the image transformation matrix using the default MATLAB parameters. With our adjusted parameters a significantly larger number of FP pairs could be obtained. Our systematic tests with other FP detectors indicated that no single detector is capable to identify sufficient number of corresponding FP pair for original FLU/VIS images using the MATLAB default parameters. Surprisingly, cropping of images to the region of interest did not improve the performance of FP detectors significantly, Fig 6.

**Table 4:** Statistics of feature point detection for successfully registered full-size, original (i.e. unfiltered) greyscale and colour-edge FLU/VIS images of Arabidopsis, wheat and maize using different FP methods: average detected FP in VIS (VIS), average detected FP in FLU (FLU), putatively matched FLU/VIS pairs (P), FLU/VIS pairs finally selected for registration (S). Statistics for manually segmented and cropped images are given in S1 Table.

	Arabidopsis VIS/FLU/P/S	Wheat VIS/FLU/P/S	Maize VIS/FLU/P/S
greyscale			
SURF	4358/243/59/5	1535/602/32/4	1089/68/8/3
FAST	4697/22/7/3	543/301/3/2	-/-/-/-
MSER	7225/87/9/3	1118/169/4/2	-/-/-/-
BRISK	7045/308/14/3	1698/455/5/2	-/-/-/-
HARRIS	0/0/0/0	-/-/-/-	-/-/-/-
MinEigen	6153/331/16/4	490/1067/4/2	-/-/-/-
KAZE	3288/99/9/3	1287/94/12/3	527/28/5/3
combined	37392/704/96/7	6000/2157/48/4	3031/166/11/3
colour-edges			
SURF	7483/2154/213/6	6361/13415/315/29	2706/548/100/32
FAST	19812/1005/43/5	3874/14704/36/7	1287/346/22/13
MSER	11943/730/35/4	2362/4177/35/5	2191/531/16/4
BRISK	27941/1226/44/4	5231/17682/44/11	2113/647/30/19
HARRIS	0/0/0/0	372/92/6/3	206/17/5/4
MinEigen	0/0/0/0	1013/886/13/4	366/79/10/7
KAZE	7014/827/36/5	2917/1340/112/62	1205/271/70/58
combined	75518/6935/364/10	21332/49901/532/104	9870/2145/233/120

Complementary to original greyscale intensity images (GS), FP registration was applied to colour-edge (CE) and manually segmented (MS) FLU/VIS images that both effectively enhance structural contrast between the background and plant regions. From Table 4 and Fig 5 it is evident that structural enhancement of CE and MS images essentially increases the number of feature points. The elevated number of FPs, in turn, results in improved success rate and overlap ratio of FP registration, Fig 6. While performance of different FP methods vary between different plant species, SURF and KAZE generally show more robust performance in comparison to other FP detectors. In addition to single FP detectors, the performance of the combined registration scheme was evaluated by which feature points of all seven detectors were merged into one single FP list. In the case of manually segmented images, combination of different feature points does not significantly improve the otherwise high number of accepted FPs and the success rate of image registration. However, under more realistic conditions (i.e. colour-edge images) the combination of feature points turns out to be pivotal for assessment of a sufficient number of FP pairs and success of multimodal image alignment.

**Fig 7:**
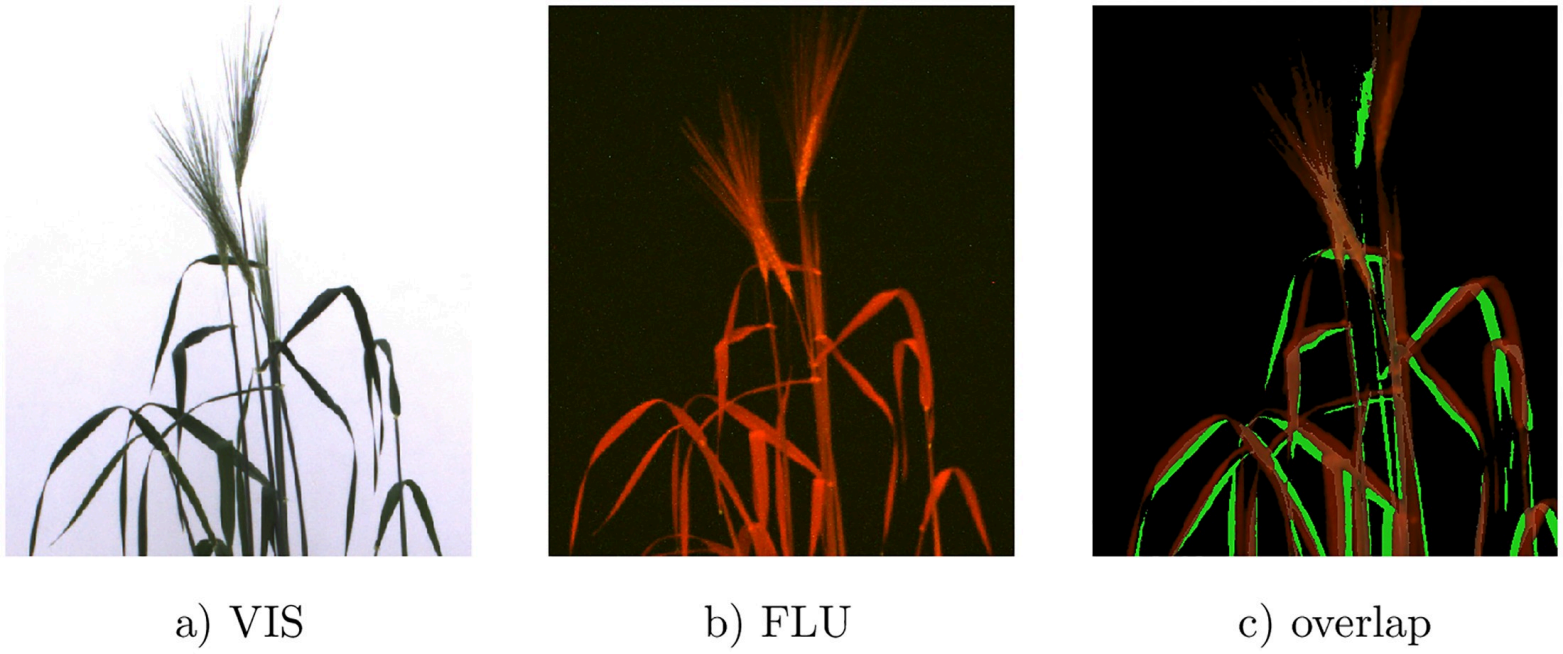
Example of non-uniform leave motion causing local misaligned of VIS (a) and FLU (b) images. Parts of the plant which are not overlapped by the FLU images are highlighted in green in the FLU/VIS overlap image (c).

A closer analysis of non-accurate image alignments (i.e. cases of *OR* ≪ 1) showed that differences between FLU and VIS can, in general, go beyond the scope of affine transformations. During the transport of large and light plants from one photo chamber to another and/or plant rotations for side view imaging, different plant organs, e.g., tillers or leaves, may move non-uniformly relatively to each other, see (Fig 7). In such cases, global affine transformation does not suffice for alignment of all plant structures resulting in a reduced accuracy (i.e. *OR* value) of image registration.

**Fig 8:**
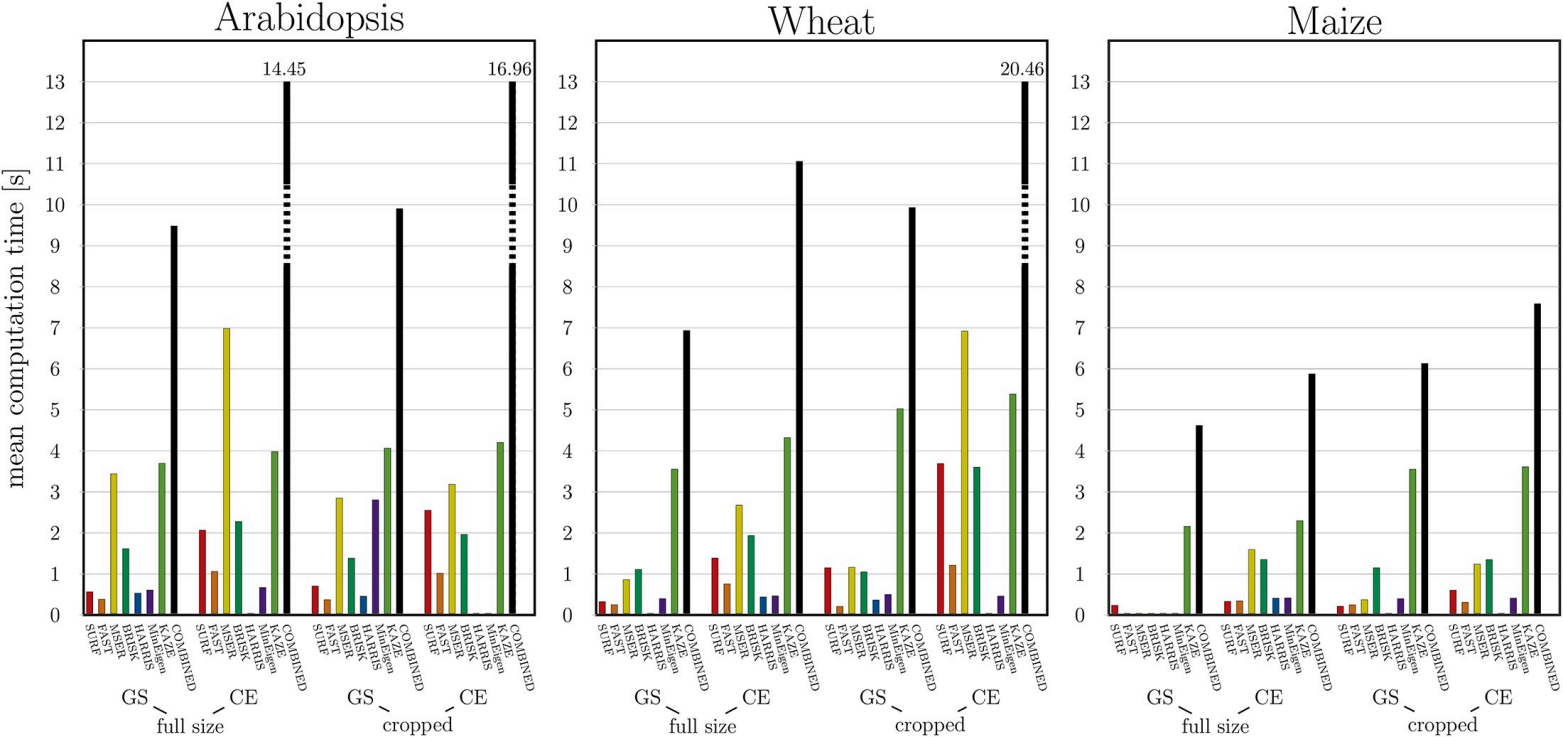
The average computation time of successful registration of original and cropped greyscale (GS) and colour-edge (CE) images of Arabidopsis, wheat and maize shoots using different FP detectors as well as their combination.

Fig 8 illustrates the average computation time of successful registration of original and cropped greyscale and colour-edge images of Arabidopsis, wheat and maize shoots using different FP detectors as well as their combination. While most single FP detectors require in average less than a second, intrinsically iterative KAZE and MSER are up to 7 times slower. The combined FP registration is, as expected, most time consuming.

**Fig 9:**
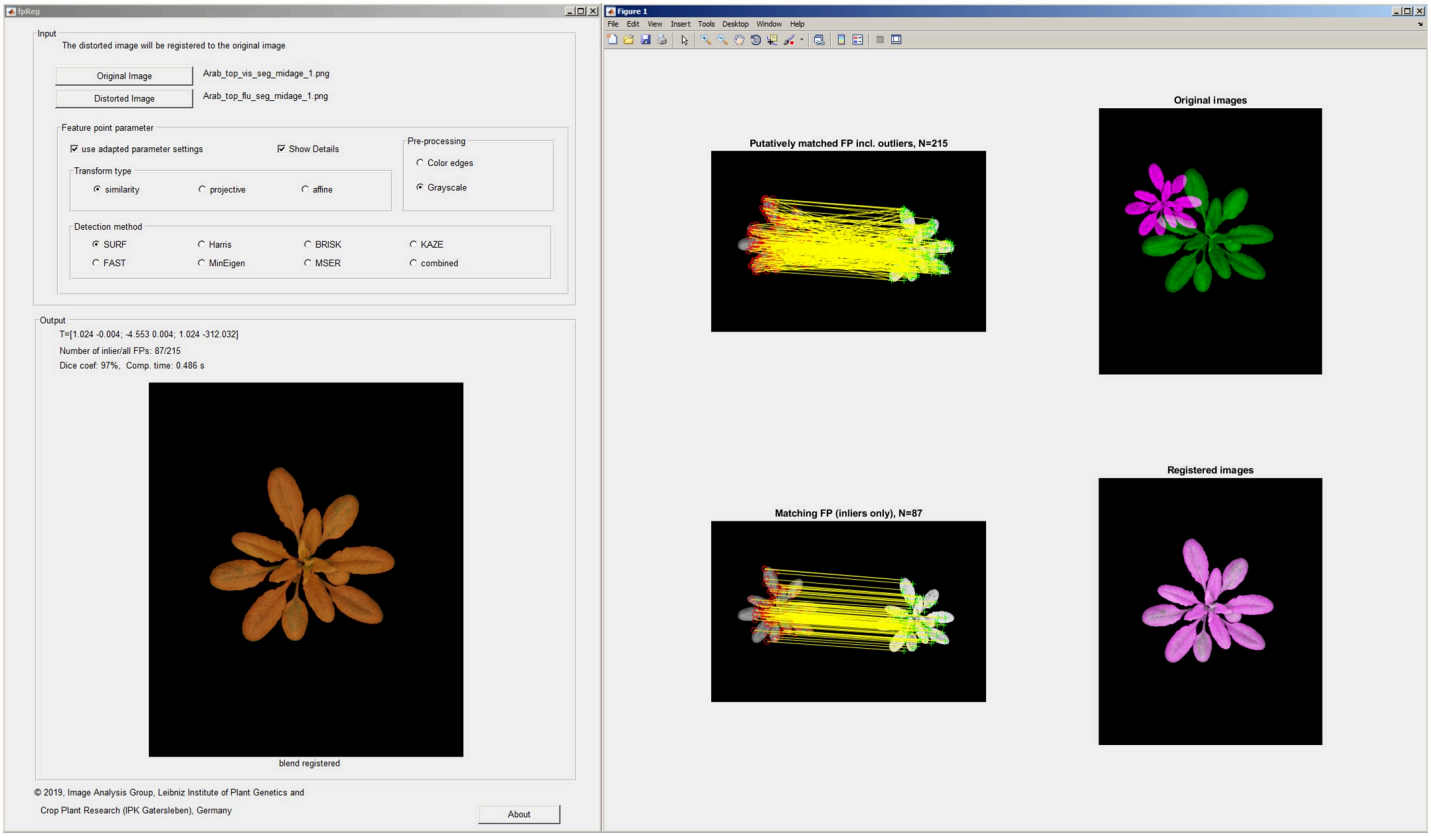
A screenshot of a GUI tool demonstrating the performance of FP algorithms for multimodal plant image registration.

A precompilied GUI tool demonstrating the peformance of different FP algorithms can be downloaded along with examples of multimodal plant images from https://github.com/ba-ipk/fpReg, see a screenshot in Fig 9.

## Discussion

Due to a number of factors including different resolution and spatial location of FLU/VIS cameras, different plant sizes and positions within the screening boxes, no universal alignment of all FLU and VIS images by a single transformation matrix is possible. Furthermore, colour and shape properties of different plants undergo considerable variations and cannot be extrapolated to mutants or other plant species. Consequently, it is not a priori clear what type of image features is most suitable for detection of relevant plant structures. Here, we show that none of the methods can be universally applied to all plant images and that combination of different registration methods is the key to achieve an exceptionally robust performance. In general, every FLU/VIS image pair has to be aligned anew. To address the problem of unknown feature detection in a very general way, seven common feature point detectors are applied and systematically evaluated according to their performance on over 1000 FLU/VIS image pairs of developing Arabidopsis, wheat and maize shoots. Our experimental results showed that large structural differences between FLU and VIS images hamper detection of a sufficient number of feature points and their pair-wise correspondences with default settings of MATLAB FP registration routines. In particular, VIS images exhibit a more complex and heterogeneous background including vertical gradient of illumination, shadows, reflections and diverse non-plant structures. Even with the adjusted set of algorithmic parameters enabling detection of more features points and their unique correspondences in comparison to MATLAB default settings the performance of FP registration on original FLU/VIS remains relatively poor. By application to original intensity images, a comparatively robust performance was observed by SURF and KAZE FP detectors. Here, we show that background elimination significantly improves success rate of FLU/VIS image registration. Improved performance of FP registration is also achieved using colour-edges instead of greyscale intensity images which suppresses modalities-specific differences of background regions and effectively makes FLU and VIS images more similar.

The major challenge of multimodal plant image registration consists in large heterogeneity and variability of structural image content which makes generalisation and optimisation of one particular FP method difficult. Plant species substantially differ by the shape and colour of leaves. Even for the same species, optical plant appearance varies dramatically in course of plant development or upon the camera position (top/side view) and rotation angle. Consequently, adjustment of FP algorithmic parameters essentially depends on particular image content and user goals. Parameters obtained in this study were tailored to a specific experimental setup and plant phenotypes, and, thus, cannot be expected to automatically produce optimal results with other image data. However, the basic approach to parameter adjustment presented in this study, i.e. increasing the number of feature points and putative FP pairs to generate a sufficient number of correspondences for calculation of reliable geometric transformations, gives a general hint of how to improve performance of multimodal image registration using FP detectors. To overcome limitations of single FP detectors, an algorithmic scheme based on combination of all seven FP methods was constructed. By integrating different feature point detectors relying on edge, corners or intensity information significant improvement of robustness and accuracy of FLU/VIS image alignment was achieved.

Our experimental results showed that FLU/VIS images can exhibit non-rigid motion due to non-uniform movements of plant tillers and leaves. This was, in particular, observed for plants with long and thin leaves like wheat and maize that experience inertial motion after translocation of carriers from one photo chamber to another or after abrupt stop of rotating carriers during the acquisition of different side views. Longer relaxation times or introduction of setups with rotating cameras should help to fix the problem of non-rigid inertial plant motion.

## Conclusion

In summary, our study shows that FP registration of appropriately pre-processed (i.e. background filtered and/or colour-edge) images can be used for automated alignment of FLU and VIS images of different plant shoots in context of high-throughput image analysis. Further investigations are required to evaluate the performance of different single and combined FP detectors on larger experiments but also on other plant species. The issue of non-rigid image motion can be principally addressed by extending the class of admissible geometric transformations to a non-rigid registration problem, however, more efficient solution would be to reduce or to avoid inertial plant movements by changing the measurement setup.

## Supporting information

S1 Table Average number of detected FPs (FLU/VIS) / putatively matched pairs / finally used feature points for successful registrations broken down to each FP methods. (PDF)

## Abbreviations

CE: colour edges
GS: greyscale
IR: infrared
FLU: fluorescence
FP: feature point
FPM: feature point method
MS: manually segmented
OR: overlap ratio
RGB: red/green/blue
SR: success rate
VIS: visible light spectrum
