# Supplementary material for: Comparison of feature point detectors for multimodal image registration in plant phenotyping
Source: PLoS One. 2019 Sep 30;14(9):e0221203. doi: 10.1371/journal.pone.0221203 (PMC6768447; doi:10.1371/journal.pone.0221203)
Supplement: S1 Table — (PDF) [file pone.0221203.s001.pdf]

Table S1: Statistics of feature point detection for successfully registered, full-size/cropped, original and hand segmented, grayscale and coluor edge VIS/FLU arabidopsis/wheat/maize images using different FP methods: average detected FP in VIS (VIS), average detected FP in FLU (FLU), putatively matched VIS/FLU pairs (P), VIS/FLU pairs finally selected for registration (S).

			Arabidopsis		Wheat		Maize	
			original VIS/FLU/P/S	hand segmented VIS/FLU/P/S	original VIS/FLU/P/S	hand segmented VIS/FLU/P/S	original VIS/FLU/P/S	hand segmented VIS/FLU/P/S
full-size	colour edges	SURF	7483/2154/213/6	604/484/73/32	6361/13415/315/29	854/879/158/91	2706/548/100/32	189/159/57/46
		FAST	19812/1005/43/5	1125/701/24/13	3874/14704/36/7	984/1325/39/21	1287/346/22/13	148/149/22/17
		MSER	11943/730/35/4	399/335/9/3	2362/4177/35/5	1269/1218/16/9	2191/531/16/4	252/227/7/5
		BRISK	27941/1226/44/4	1709/1172/38/22	5231/17682/44/11	2003/2276/78/49	2113/647/30/19	351/319/42/33
		HARRIS	-/-/-/-	49/47/4/3	372/95/6/3	628/192/15/5	206/17/5/4	87/59/12/9
		MinEigen	-/-/-/-	242/212/10/5	1013/886/13/4	952/593/28/10	366/79/10/7	131/119/17/13
		KAZE	7014/827/36/5	445/461/110/96	2917/1340/112/62	763/894/211/190	1205/271/70/58	152/150/75/72
		combined	75518/6935/364/10	4635/3488/264/168	21332/49901/532/104	7254/7161/536/368	9870/2145/233/120	1198/1081/221/187
	greyscale	SURF	4358/243/59/5	249/168/52/36	1535/602/32/4	792/649/130/78	1089/68/8/3	145/105/48/42
		FAST	4697/22/7/3	151/45/11/9	545/301/3/2	502/242/16/7	-/-/-/-	49/31/10/7
		MSER	7225/87/9/3	110/68/4/4	1118/169/4/2	283/352/9/5	-/-/-/-	58/62/6/5
		BRISK	7045/308/14/3	279/148/31/27	1698/455/4/2	804/565/36/23	-/-/-/-	117/84/20/16
		HARRIS	-/-/-/-	46/36/7/6	-/-/-/-	519/121/12/5	-/-/-/-	41/34/8/7
		MinEigen	6153/331/16/4	245/216/15/10	490/1067/4/2	784/504/22/9	-/-/-/-	103/102/17/14
		KAZE	3288/99/9/3	165/101/51/47	1287/94/12/3	29/77/7/6	527/28/5/3	11/20/7/6
		combined	37392/704/96/7	1159/723/157/127	6000/2157/48/4	3607/2376/221/124	3031/166/11/3	475/386/102/85
cropped	colour edges	SURF	6156/3925/288/5	2648/2280/183/26	14297/16944/576/12	3032/3490/323/51	5473/1365/241/41	854/716/141/65
		FAST	10338/2736/43/3	3646/2257/37/8	4746/19109/52/4	4860/6268/125/12	866/948/22/7	575/737/36/12
		MSER	4870/1156/52/3	782/741/18/3	5659/11261/67/3	3240/3545/34/5	1008/877/14/4	699/756/14/5
		BRISK	7218/1678/27/2	4827/3651/52/11	9407/29188/83/5	7599/9648/158/25	1599/1477/36/12	1181/1321/68/28
		HARRIS	-/-/-/-	162/262/6/3	-/-/-/-	1287/982/25/3	-/-/-/-	152/290/10/3
		MinEigen	-/-/-/-	681/829/15/3	161/962/3/2	2558/3121/53/5	192/154/7/3	476/660/24/6
		KAZE	6529/2032/37/6	1834/1348/164/107	6545/3018/122/31	3119/3450/300/136	1550/614/126/83	400/531/103/77
		combined	60440/17817/482/9	13947/11458/472/145	41554/81332/897/46	25333/29552/1001/225	10142/4917/428/139	4159/4828/387/188
	greyscale	SURF	2244/561/86/5	980/511/90/35	3750/3481/103/4	2061/1798/198/51	552/189/12/3	463/324/85/49
		FAST	4069/39/13/3	391/110/19/12	363/688/5/2	1432/702/23/3	-/-/-/-	76/102/8/4
		MSER	4881/33/5/2	121/61/4/3	1420/285/6/2	800/523/10/3	414/124/2/2	121/110/5/3
		BRISK	2230/100/8/3	487/173/29/23	1544/1020/6/2	2552/1479/56/16	535/65/2/2	187/182/21/12
		HARRIS	1018/308/12/2	134/315/7/4	410/47/3/2	1347/575/18/3	-/-/-/-	95/363/6/3
		MinEigen	17290/5345/218/4	707/880/18/6	1463/390/6/2	2081/2302/28/3	448/41/5/2	399/525/15/5
		KAZE	1620/95/8/3	343/140/56/48	1370/118/8/2	313/176/19/9	498/29/5/2	33/28/9/8
		combined	29736/3977/240/9	2786/2047/189/103	9356/5053/128/4	10760/7386/348/79	1647/307/18/3	1274/1457/137/73
